# Diagnosis, treatment, and outcome prediction of non-convulsive status epilepticus in unconscious patients in intensive care units

**DOI:** 10.1186/s42466-025-00435-7

**Published:** 2025-10-24

**Authors:** Laurent Maximilian Willems, Isabelle Beuchat, Urs Fisch, Raoul Sutter, Christoph Kellinghaus, Adam Strzelczyk

**Affiliations:** 1 Epilepsy Center Frankfurt Rhine-Main, University Medicine Frankfurt, Theodor-Stern-Kai 7, 60596 Frankfurt am Main, Germany; 2 Department of Neurology, University Medicine Frankfurt, Frankfurt am Main, Germany; 3 Department of Neurology, Centre Hospitalier Universitaire Vaudois, University of Lausanne, Lausanne, Switzerland; 4 Department of Neurology, University Hospital Basel, Basel, Switzerland; 5 Intensive Care Unit, University Hospital Basel, Basel, Switzerland; 6 Medical Faculty, University of Basel, Basel, Switzerland; 7 Department of Neurology, Klinikum Osnabrück, Osnabrück, Germany; 8 School of Health and Wellbeing, University of Glasgow, Glasgow, UK

**Keywords:** Seizure, Epilepsy, Coma, Intensive care, Loss of consciousness, EEG

## Abstract

**Background:**

Non-convulsive status epilepticus (NCSE) is a common definitive or exclusion diagnosis in patients with disorders of consciousness (DOC) on neurological or interdisciplinary intensive care units (ICU). Special expertise is required to ensure reliable diagnosis, targeted therapy management, and individual prognostication, particularly as NCSE is identified based solely on clinical and electroencephalographic findings.

**Main body:**

This narrative state-of-the-art review compiles and critically discusses the existing literature on various aspects of NCSE. We focus on the reliable initial diagnosis and further monitoring of NCSE using the Salzburg criteria and the 2HELP2B score, therapy options beyond current guideline recommendations, and prognosis assessment using established scores and metrics, such as STESS, SACE, EMSE, and END-IT. With compact tables and clear illustrations, comprehensive insights are presented in a concise structure to provide clear guidance for daily practice.

**Conclusion:**

NCSE is a common and complex disease entity observed in the ICU that requires dedicated and specialised diagnostics, therapy, monitoring, and outcome assessment. Evidence-based recommendations are now available for each of these critical processes to guide caregivers and relatives. However, the availability of continuous (cEEG), quantitative (qEEG) electroencephalography in the ICU and expertise in its interpretation are limiting factors in many clinical settings. This problem is becoming increasingly pronounced due to the reduced or complete lack of reimbursement for c/qEEG in the context of intensive care medicine in many countries.

## Main part

### Background

Non-convulsive status epilepticus (NCSE) represents a complex and often underdiagnosed neurological emergency characterised by ongoing epileptic activity without prominent motor symptoms. In contrast to convulsive status epilepticus (CSE), NCSE presents with subtle or atypical clinical signs, such as altered mental status, confusion, or behavioural changes, making prompt recognition particularly challenging in emergency and intensive care unit (ICU) settings [55]. The diagnosis of NCSE relies primarily on electroencephalography (EEG); however, the interpretation of EEG patterns can be complex and requires specialised expertise to distinguish epileptic activity from other encephalopathic processes or the ictal–interictal continuum (IIC) [70, 92, 104]. This diagnostic challenge is compounded in emergency scenarios where rapid decision-making is crucial, often under conditions of limited EEG availability or expertise [55]. The heterogeneity of NCSE presentation necessitates a high index of suspicion and continuous EEG monitoring to prevent misdiagnosis and subsequent neurological deterioration [59]. Therapeutically, NCSE management is associated with significant difficulties. Specific evidence for the treatment of NCSE is limited, as common guidelines mostly pertain to CSE. However, there are established treatment concepts for NCSE in the initial phase and for refractory or super-refractory progression [28].

The aim of this scoping review is to summarise and discuss specific demographic, aetiologic, diagnostic, and therapeutic aspects of NCSE based on the most recent literature.

## Prevalence and aetiologies

NSCE is increasingly recognised as an underdiagnosed cause of persistent unconsciousness in critically ill patients. The true prevalence among unconscious patients remains difficult to determine due to variability in monitoring protocols and definitions. However, studies using continuous EEG (cEEG) suggest that NCSE may be present in 8%–37% of patients with unexplained coma [15, 80].

In a prospective neurological ICU study, electrographic seizures (NCSE/and non-convulsive seizures) were detected in 21% of patients with altered mental status, excluding those with anoxic injury. Among the NCSE group, focal seizures predominated (86%), and subtle clinical signs such as facial twitching or eye deviation were present in 50% of cases [51]. Significant risk factors included prior epilepsy, intracranial tumours, meningitis or encephalitis, and imaging findings such as encephalomalacia. NCSE was associated with increased mortality (31% vs. 14% in non-NCSE), and EEG monitoring led to changes in antiseizure drug therapy in 39% of cases [51]. In highly vulnerable post-anoxic patients, the prevalence of NCSE may be even higher. For instance, NCSE was reported in up to 34% of post-cardiac arrest patients, reinforcing the need for EEG surveillance in unconscious populations [82].

More recent data from hospital-based studies further highlight the clinical burden of NCSE. In a population-based study, an annual NCSE incidence of 12.1 per 100,000 adults was observed, using the operational definition of status epilepticus (SE) published by the International League Against Epilepsy (ILAE) in 2015, outlined below. Notably, patients with NCSE and impaired consciousness had a substantially higher in-hospital mortality rate (33%) compared to those who remained awake (8%) [58]. These findings underscore the prognostic importance of both seizure activity and level of consciousness.

In a prospective study, the true prevalence of NCSE in a monocentric cohort of 469 patients was 11% based on final clinical diagnosis. The most frequent aetiologies were intracranial tumour (19%), cerebral anoxia (16%), known epilepsy (13%), and cerebrovascular disease (13%). The findings highlight that encephalopathy (including metabolic, septic, and post-anoxic) often mimics NCSE and contributes to diagnostic uncertainty [115].

To standardise the assessment and classification of SE, including NCSE, the ILAE published a revised classification in 2015 [111]. This classification distinguishes five aetiological categories: acute symptomatic, remote symptomatic, progressive, genetic/idiopathic, and unknown. Acute symptomatic aetiologies refer to seizures occurring in close temporal proximity to an acute brain insult, such as stroke, trauma, metabolic disturbance, or central nervous system infection. Remote symptomatic causes relate to prior brain injuries that are no longer considered active, such as previous strokes or traumatic brain injury. Progressive aetiologies involve ongoing or worsening neurological disorders, including brain tumours or neurodegenerative diseases. Idiopathic or genetic causes are rare in adults and are more often seen in paediatric patients. Finally, some cases remain of unknown aetiology, even after extensive evaluation [111].

NCSE aetiologies in unconscious patients reported in the literature are displayed in Table 1**,** organised by clinical categories and estimated prevalence.

**Table 1:** Common aetiologies of non-convulsive status epilepticus (NCSE) in unconscious patients

NCSE Aetiology	Specific aetiologies	NCSE Prevalence in %
Structural Brain Injury	Ischemic stroke Intracerebral haemorrhage Traumatic brain injury	10–20% [15, 80]
Anoxic Brain Injury	Post-cardiac arrest Respiratory failure with hypoxia	Up to 34% [82]
Metabolic Disturbances	Hepatic encephalopathy Uremic encephalopathy Hypoglycaemia Hyponatraemia	5%–15% [105]
Drug-Related Causes	Withdrawal from antiepileptic drugs Toxic effects (e.g., beta-lactams, quinolones) Benzodiazepine or alcohol withdrawal	~ 10% [1, 105]
Pre-Existing Epilepsy	Poor medication adherence Breakthrough seizures	15%–20% [1]
Infectious/Inflammatory	Encephalitis (viral, autoimmune) Sepsis-associated encephalopathy	~ 5%–10% [80, 105])
Neoplastic	Primary or metastatic brain tumours Paraneoplastic syndromes	Rare, < 5% [5]
Other/Cryptogenic	Unknown causes Neurodegenerative conditions	10%–20% [105]

### Diagnosis and monitoring

NCSE is characterised by prolonged or repeated epileptic activity without prominent motor signs, manifesting instead as altered mental status. Timely diagnosis is crucial but difficult due to the absence of convulsions. Indeed, approximately 90% of ictal EEG patterns lack overt clinical signs, justifying the terms “non-convulsive” or “subclinical”. Conversely, around 75% of abnormal movements in the ICU are not seizure-related and stem from other causes [25].

When NCSE is suspected, clinicians must decide between cEEG monitoring and shorter routine or repeated EEG (rEEG) recordings [122, 123]. Also reduced point-of-care EEG systems (POC-EEG) can be used for the diagnosis of NCSE. Here, but also in cEEG or rEEG, artificial intelligence (AI) can help speed up and simplify diagnostics. [26]. In addition to its clinical benefits, POC-EEG has also been shown to offer economic benefits by avoiding unnecessary hospital transfers, shortening hospital stays, and reducing direct costs in acute care. Nevertheless, the use of POC EEG is not widespread and is usually only established in specialized centers [33].

Continuous EEG detected seizures in 15.7% of ICU patients in a randomised controlled trial, compared to just 4.4% when using repeated 20 min rEEGs. Treatment adjustments were also more frequent in the cEEG group (21.1% vs. 11.5%). However, long-term outcomes—mortality and functional recovery—were similar between groups [83]. To assist in determining seizure likelihood and appropriate EEG duration, systems like the 2HELPS2B score [94] (Table 2) and the TERSE (time-dependent electro-clinical risk stratification) algorithm [14] have been developed, incorporating early EEG features and clinical context. A pragmatic, risk-adapted approach to guide EEG duration is summarised in Table 3.

**Table 2:** The 2HELPS2B Score

Acronym	EEG finding	Points
*Scoring*		
2H	GRDA, LRDA, BIPD, LPDs or GPDs with a frequency > 2 Hz	1
E	Epileptiform discharges	1
L	LPD, LRDA or BIPDs	1
P	Plus modifiers (superimposed rhythmic, sharp, or fast activity)	1
S	Seizures (acute or remote prior seizures)	1
2B	BIRDs	2

Total points	Seizure / status epilepticus risk (%)
*Interpretation*	
0	3
1	12
2	34
3	52
4	71
5	84
6	92

GRDA, generalised rhythmic delta activity; LRDA, lateralised rhythmic delta activity; BIPD, bilateral independent periodic discharges; LPD, lateralised periodic discharges; GPD, generalised periodic discharges; BIRDS, brief potentially ictal rhythmic discharges

Adapted from [94]

**Table 3:** Recommended EEG recording length based on clinical context and the 2HELPS2B score

Step:		Diagnostic and therapeutic approach				
1	Context	Terminated seizure / SE	Ongoing SE	Unexplained DOC	Risk of delayed cerebral ischaemia (e.g., SAH)	Hypoxic-ischaemic encephalopathy
2	Start with	rEEG	cEEG	rEEG	cEEG (with qEEG)	rEEG Repeat after 24-48 h
3	2HELPS2B < 2 and no seizure / SE	rEEG Repeat after 24-48 h	cEEG Stop after 3–6 h	rEEG Repeat after 24-48 h	Continue cEEG for up to 10–14 days	rEEG Repeat after 24-48 h
	2HELPS2B ≥ 2 and ongoing seizures or SE	Escalate therapy Switch to cEEG for 24-48 h	Escalate therapy Continue cEEG for 24-48 h	Escalate therapy Continue cEEG for 24-48 h	Escalate therapy Continue cEEG for 10-14d	Escalate therapy^a^ Switch to cEEG for 24-48 h

Modified from [21, 94, 95]

EEG, electroencephalography; rEEG, repetitive EEG; cEEG, continuous EEG; DOC, disorders of consciousness; SAH, subarachnoid haemorrhage; SE, status epilepticus; qEEG, quantitative EEG

^a^Escalation of therapy and EEG monitoring only if multimodal assessment compatible with favourable prognosis

The American Clinical Neurophysiology Society (ACNS) provides a comprehensive framework for describing ICU EEGs. Its Critical Care EEG Terminology offers standardised definitions for seizures and SE in critically ill patients [38]. Notably, it introduced formal definitions for electrographic seizures and electrographic SE. Electrographic seizures are defined as epileptiform discharges at a frequency of ≥ 2.5 Hz lasting ≥ 10 s. Electrographic SE is defined as seizure activity lasting ≥ 10 min or occupying ≥ 20% of any 60-min EEG epoch. Importantly, the 2021 ACNS update acknowledges ambiguous cases within the IIC: EEG patterns that do not fully meet seizure criteria but improve with anti-seizure medication (ASM) are termed “possible” seizures or status [38].

**Fig. 1:**
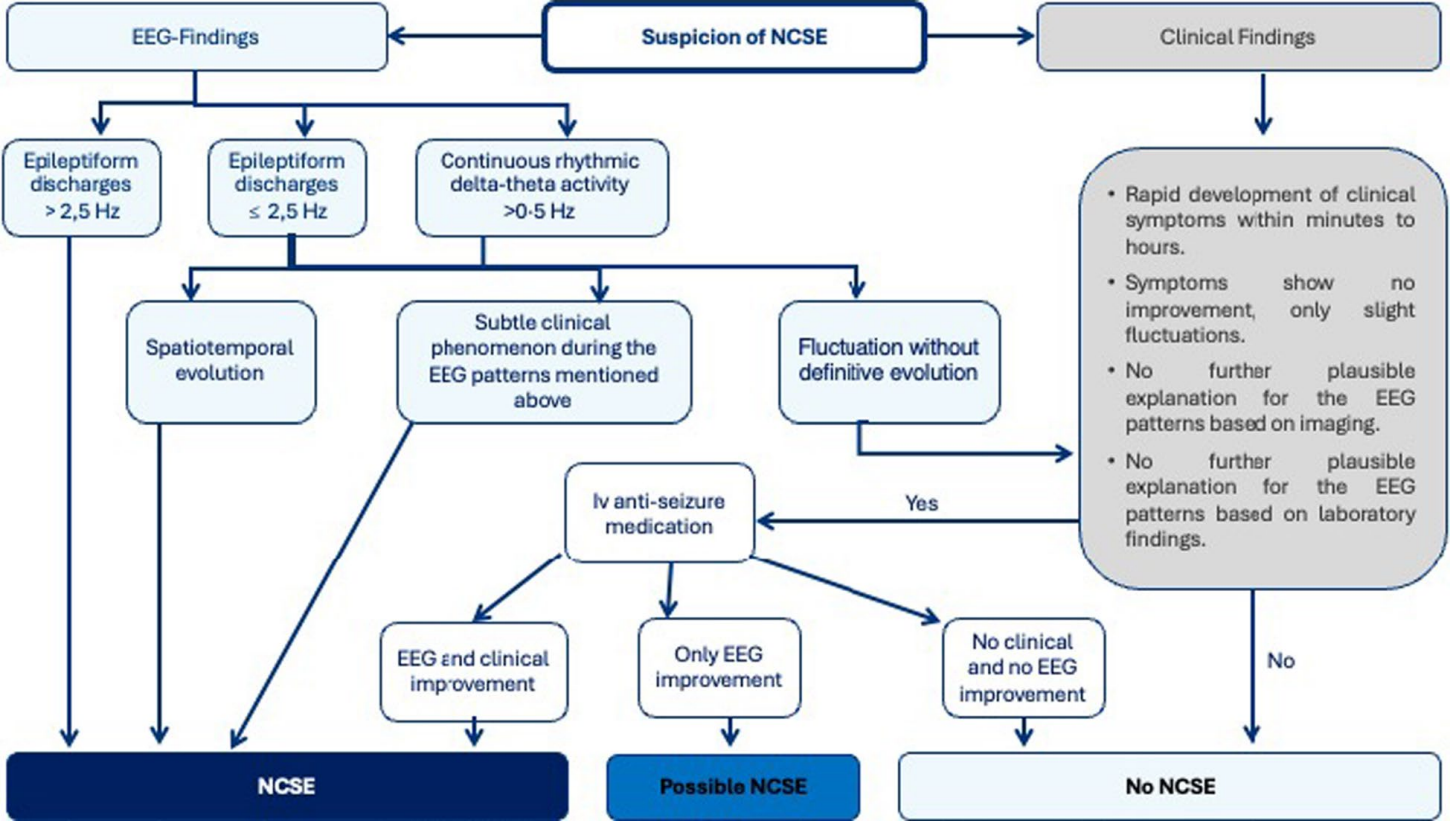
Salzburg Criteria: Schematic overview and flow chart of the Salzburg Criteria for the diagnosis of non-convulsive status epilepticus (NCSE) based on clinical and electroencephalographic findings resulting in a definite, possible, or rejection of the diagnosis

The Salzburg Criteria, developed in 2013 by an expert panel and officially published in 2016, provide a standardised diagnostic framework for NCSE (Fig. 1) [54, 56, 57]. These criteria were inspired by earlier frameworks from Young and Kaplan [43, 125] and align closely with the ACNS nomenclature [38]. Diagnosis is based on specific electrographic features interpreted within their clinical context [54, 56, 57]. The Salzburg Criteria identify three core EEG patterns, any of which support diagnosis in patients without prior epileptic encephalopathy if present for ≥ 10 s **(**Fig. 1**)** [54, 56, 57]. These patterns are epileptic discharges > 2.5 Hz, or ≤ 2.5 Hz with at least one secondary feature (e.g., evolution, subtle clinical signs, or ASM response), and rhythmic delta or theta activity > 0.5 Hz with at least one secondary feature [127].

In patients with pre-existing encephalopathy, an additional criterion is required to improve specificity: either a clear deterioration in EEG relative to baseline or clinical/EEG improvement following ASM administration [54, 56, 57]. In a validation cohort, the criteria demonstrated a sensitivity of 97.7%, specificity of 89.6%, and overall diagnostic accuracy of 92.5%, with inter-rater agreement indicating excellent reproducibility (κ = 0.87) [57]. External validation in broader populations has shown lower sensitivity but higher inter-rater reliability, with authors emphasising the diagnostic challenges posed by encephalopathic EEGs [32]. Later studies have also noted that these criteria may not be applicable to post-anoxic patients, in whom low-frequency generalised periodic discharges may represent SE [50]. Despite these limitations, the Salzburg Criteria remain a robust diagnostic tool, particularly when combined with ACNS EEG terminology and applied within a structured electroclinical decision-making framework [38, 54, 56, 57].

Until recently, the evaluation and classification of typical NCSE EEG findings was the responsibility of experienced examiners. For several years now, the potential of different computer-based tools for detecting NCSE and associated EEG findings has been increasingly exploited, and existing machine learning and AI tools have been developed and continuously improved [18]. A systematic review and meta-analysis to evaluate the clinical impact and accuracy of artificial intelligence in EEG for the early detection of NCSE demonstrated the high potential of AI-supported evaluation, but also highlighted the existing hurdles and problems of this technology, which is still in its developmental stages [31]. Nevertheless, several software solutions for quantitative EEG analysis (qEEG) are available on the market and are already in further clinical use in the context of NCSE [85].

### Standard therapy according to guidelines

The management of NCSE is focused on stabilising vital functions, halting seizures, and identifying and treating underlying causes. Rapid initiation of first-line therapy, with escalation as needed, is critical to prevent treatment-refractoriness [12, 44, 47]. The following sections outline escalating antiseizure treatment and highlight the underexplored areas of tracheal intubation timing and target titration parameters for continuous intravenous anaesthetic drug (CIVAD) therapy. Beyond pharmacologic choices, structured emergency systems and individualised seizure action plans are equally critical for treatment success, but are beyond the scope of this review.

Upon stabilisation, and while simultaneously investigating underlying causes, guidelines uniformly recommend benzodiazepines (BZDs) as first-line antiseizure agents due to their efficacy, safety profile, and availability [11, 30, 63, 77, 120]. Lorazepam, midazolam, and diazepam are the most recommended, while clonazepam is very rarely used (Table 4). The choice depends on availability, storage conditions, cost, and provider familiarity, rather than pharmacokinetics. Although intravenous (IV) applications are preferred, alternative routes (intramuscular, intranasal, rectal, or buccal) are available and may be more suitable for first responders or untrained caregivers [19, 35, 48, 91]. Repeated dosing is often advised if initial attempts fail, though clinicians must monitor for adverse effects like respiratory depression and sedation, which may necessitate airway management [49],Sutter, Tisljar, et al., 2019). First-line treatments are often underdosed, which may be explained by fear of complications [2, 47].

**Table 4:** Treatment options for non-convulsive status epilepticus according national and international guidelines

Therapeutic option		Application	Initial dosing			Maintenance dosing			Comment and caveat
Substance and Abbreviation		Route	Loading (mg/kg)	Maximal (mg)	Per 75 kg BW (mg)	Single dose (mg) or perfusor rate (mg/kg/h)		Schedule	
First-line treatment									
Lorazepam	LZP	IV buccal	0.1	4	4	0.5 – 1		BID, TID	Re-application after 10 min possible
Diazepam	DZP	IV buccal rectal intranasal	0.2 5 – 15^1^ 0.2 – 0.5 5—20	10 15 20 20	10 10 20 20				
Midazolam	MDZ	IV buccal intranasal intramusuclar	0.2 0.3 – 0.5 0.2 5 – 10	20	15				
Clonazepam	CZP	IV	0.015	1	1				
Second-line treatment									
Levetiracetam	LEV	IV	40 – 60	4,000	4,000	2,000	BID		Adjustment according to serum level
(Fos)Phenytoin	PHT	IV	20^2^	1,500^2^	1,500	250	BID, TID		
Valproate	VPA	IV	20 – 40	3,000	2,500	400	TID		
Lacosamide	LCM	IV	5	400	400	200	BID		
Third-line treatment									
Midazolam	MDZ	IV	0.2			0.05 – 2^3^	CONT		Only to be used in ICU settings
Propofol	PPF	IV	1 – 2			1.5 – 4.5^3^	CONT		
Pentobarbital	PB	IV	5			0.5 – 5^3^	CONT		
Thiopental	TP	IV	1 – 5			0.5 – 5^3^	CONT		
Ketamine^4^	KET	IV	0.5 – 3			1.5 – 10^3^	CONT		

IV, intravenous, ICU, intensive care unit, BID, two times per day, TID, three times per day, CONT, continuous infusion, BW, bodyweight

^1^ mg

^2^phenytoin equivalents

^3^ mg/kg/h

^4^treatment option with limited evidence

Second-line antiseizure treatment relies on IV formulations of non-BZD ASMs (Table 4) [11, 30, 63, 120]. The Established Status Epilepticus Treatment Trial (ESETT) showed equipoise for IV valproic acid, levetiracetam, and fosphenytoin [45]. There is growing evidence for the similar efficacy of IV lacosamide (LCM) and brivaracetam (BRV), although larger prospective trials are lacking [34, 42, 69, 89, 101, 102]. German Guidelines now include LSM as a second-choice second-line drug with caution advised due to potential cardiac side effects, reflecting the therapeutic standard of many epilepsy centres and emergency facilities [77]. One trial failed to demonstrate a benefit of the immediate addition of a second-line ASM after BZDs; however, an approach which does not require waiting for the effects of first-line agents to diminish before initiating ASMs once SE is confirmed seems legitimate to reduce the risk of rebound SE or seizures. Many treatment algorithms have been developed for convulsive SE and are applied to NCSE, but some researchers have recommended the development of more nuanced approaches [11, 30, 63, 120]. NCSE is an overarching term representing a heterogeneous group. These guidelines advocate for considering SE semiology, outcome-relevant comorbidities, and patient directives when balancing potential treatment benefits against treatment-associated risks [7, 8, 77, 103, 106]. According to the Salzburg criteria, patients with epileptiform discharges < 2.5 Hz or rhythmic activity > 0.5 Hz may fulfil a possible or definite NCSE diagnosis, depending on their EEG and clinical response to IV ASM. A recent experts’ panel proposed operational criteria for EEG and clinical responses. The panel agreed that either BDZs or non-BDZs can be used as an initial IV ASM. They proposed that non-BZDs should be used first in patients with impaired alertness or at risk of respiratory depression, highlighting a more nuanced approach for NCSE treatment [55]. Following unsuccessful second-line therapies, the evidence for further management of treatment-refractory SE (RSE) becomes increasingly limited. If a single ASM or a combination thereof fails to terminate SE, mechanical ventilation and CIVAD—such as with midazolam, propofol, or barbiturates—should be considered (Table 4). In recent years, clinicians have tended to use the first two options, although direct comparisons are difficult due to selection biases of retrospective studies [4]. Intubation should be reserved for cases involving respiratory failure, inadequate airway protection, or the need for CIVAD-induced coma. Emergency intubation carries risks, including hemodynamic instability, hypoxemia, and cardiac arrest [84]. A secondary analysis of the ESETT trial revealed variability in intubation rates, often influenced by site-specific protocols rather than clinical necessity [78]. The benefit of ultra-early intubation remains controversial, with conflicting data from different retrospective studies [6, 16, 17, 112].

In a multicentre retrospective cohort study, 72% of RSE patients were successfully treated without intubation. These patients were older, on average, and acute aetiologies, convulsive SE, and NCSE with stupor or coma were less common. Importantly, non-intubation was not associated with poorer outcomes [8]. These data support the opinion of many experts that most focal SE with preserved consciousness and specific generalised SE syndromes, such as absence SE, can be managed without the need for intubation [107].

Once CIVAD-induced coma is initiated, optimal duration and titration strategies of CIVAD remain underexplored. Seizure cessation and burst suppression are recommended equally by guidelines [11, 30, 63]. However, the evidence supporting burst suppression is limited and may reflect a period when cEEG was scarce and easily identifiable EEG patterns were preferred for intermittent monitoring [22, 36]. Indeed, the idea that burst suppression is a stable pattern is a misconception [3].

Several studies have investigated coma depth in RSE but not exclusively refractory NCSE [4, 22]. The largest retrospective analysis to date did not show differences in short-term functional outcomes when comparing RSE patients reaching seizure cessation or burst suppression [23]. However, another study from the same cohort demonstrated that any CIVAD dose escalation was associated with decreased mortality without increasing the proportion of survivors with unfavourable outcomes, advocating for active CIVAD management [24]. The heterogeneity and low quality of evidence support individualised and EEG-guided CIVAD management rather than rigid adherence to historical protocols.

### Advanced and experimental therapeutic options

The strongly evidence-based recommendations of national and international guidelines are sufficient for the treatment of most patients with NCSE. However, there are individual cases, involving a lack of response or other medical or ethical factors, in which less evidence-based treatment options must be considered. In this context, the evidence-based prior stages of therapy described in Sect. 2.2 should not be bypassed under any circumstances. Existing therapies with ASM should be continued and benzodiazepines should either be administered continuously or repetitively [77]. Due to the frequent occurrence of NCSE in older patients and those with pre-existing conditions, restrictions concerning CIVAD often necessitate treatment beyond guideline recommendations [121, 130].

Prior to discussing individual options for escalating treatment in (super)refractory NCSC in more detail, it is important to emphasize that the available evidence for the effectiveness and tolerability of these treatment options is extremely limited and mostly limited to single case reports, case series, or studies involving small, mostly retrospective or prospective cohorts [121]. Since all of the treatment options described below are off-label, treatment should only be carried out after an individual risk–benefit assessment and consultation with experienced practitioners. Larger prospective randomized controlled trials are urgently needed to improve the level of evidence in this regard. Rapidly titratable ASM, administered orally or intravenously, is the primary treatment option in these cases [121]. Already included in some guidelines, LCM is notable as it has the largest evidence base for the treatment of NCSE, is easy to titrate, and shows negligible interactions with other ASMs [52, 67, 88, 97, 101, 102]. There is also evidence in the literature for the effectiveness of the SV2A modulator BRV [61, 69, 89, 101, 102], the AMPA modulator perampanel (PER) [39, 75, 98], the sodium channel blocker oxcarbazepine (OXC) [46], and the polymechanistic ASMs topiramate (TPM) and zonisamid (ZNS) [20, 40, 41, 110]. For severe or (super)therapy-resistant cases in particular, the GABAergic stiripentol (STP)—primarily approved for the treatment of Dravet syndrome—has been demonstrated to be effective both in murine and human studies [29, 66, 93, 99, 113, 114]. However, due to the high costs involved, STP is not yet widespread in the treatment of advanced NCSE [76]. Fenfluramine may represent a further novel option for developmental and epileptic encephalopathies presenting with SE; however, so far, only a limited number of case reports have been published [96].

In addition to the option of oral or IV ASMs, ketogenic diet therapy (KD) is a feasible, simple to implement, well-tolerated and evidence-based option in the treatment of NCSE [60]. In most cases, a KD ratio of 4:1 of fats to nonketogenic proteins and carbohydrates is used; this formulation is available as a ready-to-use solution for feeding tubes [60, 62], and can also be initiated or maintained via IV administration [100]. Whenever possible, KD should be closely supervised by a nutritionist to prevent possible metabolic imbalances and to optimise outcomes [121]. In addition to KD, magnesium sulfate (MgSO4) saturation and supplementation is a potential supportive treatment option in NCSE [87].

If an autoimmune origin of NCSE is suspected or confirmed, immunosuppressive therapies should also be considered in the early stages. In addition to steroid pulse (SP) therapy, IV immunoglobulins (IVIG) or plasma exchange (PLEX) are also available options [72, 124]. While animal studies and small case series gave promising indications of the potential benefits of neurosteroids, such as allopregnanolone or ganaxolone [65, 74, 118], these could not be confirmed in larger randomised controlled studies in humans [86, 119]. The use of neurosteroids in the treatment of super-refractory SE therefore lacks any clinically robust evidence.

If maximal medical therapy is desired and all other therapeutic methods have been exhausted, further invasive options are available for the treatment of NCSE, but these have a low level of evidence [121]. As with the treatment of super-refractory SE, individual case reports suggest the use and effectiveness of volatile anaesthesia procedures. These approaches are already established in many ICUs, as well as for epilepsy surgery or invasive neurostimulation in NCSE [13, 53, 68]. However, the use of these very low-evidence procedures should be reserved for individual cases and should only be performed at appropriately specialised centres after interdisciplinary consideration [121]. All available and evidence-based treatment options beyond the standard guideline recommendations are summarised in Table 5.

**Table 5:** Treatment options for non-convulsive status epilepticus beyond guideline recommendations

Therapeutic option		Application		Dosing		Comment and caveat
Substance and Abbreviation		Route	Regimen	Loading (mg)	Maintenance (mg/d)	
Antiseizure Medication^1^						
Brivaracetam	BRV	IV, PO	BID	200	100 – 200	Psychobehavioural changes
Oxcarbazepine	OCX	PO	BID	600–1200	1200–1800	Hyponatraemia
Perampanel	PER	PO	QD	8–12	8–12	Dizziness, vertigo
Stiripentol	STP	PO	BID	1000	2000–4000	High treatment costs
Topiramate	TPM	PO	BID	400	400–600	Hyperammonaemia, metabolic acidosis
Zonisamide	ZNS	PO	BID	300	300–600	Ataxia, rush
Anesthetics^1^						
Isoflurane	ISO	IP	CONT	MAC 0.2–0.5%		Malignant hyperthermia, increased intracranial pressure
Nutritional therapy, Supplementation^1^						
Ketogenic diet	KD	PO	CONT	Ratio 4:1^2^		Metabolic acidosis, hyperlipidaemia, electrolyte disbalances
Magnesium	MgSO_4_	IV, PO	CONT	4000	2000–6000	Cardiac arrhythmia
Immunotherapies^1^						
Steroid pulse	SP	IV, PO	QD	1000	1000	If autoimmune aetiology assumed or confirmed
Immunoglobulins	IVIG	IV	TID	0.4^3^		If autoimmune aetiology assumed or confirmed
Plasma exchange	PLEX	IV	INT	n.a	n.a	If autoimmune aetiology assumed or confirmed

IV, intravenous administration; PO, oral intake, IP, intrapulmonary administration, BID, twice per day, QD, once per day, TID, three times per day, CONT, continuous infusion, INT, intermittent therapy, MAC, minimal alveolar concentration

^1^All treatment options mentioned here have a low level of evidence, which is mostly based only on case reports, case series, or smaller retrospective or prospective studies. They should therefore only be used after appropriate risk–benefit assessment and individual case analysis

^2^ratio of fat vs. combined protein and carbohydrate intake

^3^g/kg/d for 5 days

### Prognostication and outcome

Accurate prognostication in NCSE is essential for clinical decision-making, patient counselling, and therapeutic planning [79]. While the heterogeneity of NCSE presentations and underlying aetiologies makes outcome prediction challenging, several validated scoring systems have been developed to assist in risk stratification [126]. Overall, case-fatality has not significantly changed in recent decades. It is predominantly determined by baseline characteristics such as advanced age, life-threatening underlying aetiologies, and medical comorbidities, rather than by specific treatment modalities, which appear to exert relatively modest independent effects on survival [71, 79].

The Status Epilepticus Severity Score (STESS) was one of the first widely adopted tools, incorporating four readily available clinical parameters at presentation: age (≥ 65 years: 2 points), history of seizures (none: 1 point), seizure type (generalised tonic–clonic: 1 point, NCSE-coma: 2 points), and consciousness (stupor/coma: 1 point) [81]. The STESS can reliably identify patients who will survive, with studies showing high negative predictive values for mortality [9, 10, 90]. However, operational failures of the STESS have been identified, with false-positive rates of 51% in some cohorts, particularly affecting patients with less fatal aetiologies and lower comorbidity burdens (Sutter, [90]). The Epidemiology-Based Mortality Score in Status Epilepticus (EMSE) was developed to address some limitations of the STESS by incorporating aetiology-specific mortality data [54, 56]. EMSE demonstrated better performance than the STESS in several validation studies [9, 10, 116]. For NCSE-specific populations, the newly developed Salzburg Criterion A2 (SACE) score represents a more targeted approach [64]. The SACE score integrates Salzburg NCSE EEG criteria (i.e., the Salzburg criterion A2 of typical ictal evolution) with established clinical parameters such as age, history of seizure, and level of consciousness. The Salzburg NCSE criterion A2 of ictal morphological, spatial, and temporal evolution is associated with in-hospital survival [64]. The END-IT (encephalitis, NCSE, diazepam resistance, imaging abnormality, and tracheal intubation) score was originally developed for convulsive SE in a young, Chinese population with high prevalence of encephalitis cases [27], but has since shown utility in outcome prediction. For long-term survival prediction, the Age-Consciousness-Duration (ACD) score addresses limitations of existing tools that focus primarily on in-hospital mortality [73]. The ACD score incorporates age at onset, level of consciousness at admission, and duration of SE [73]. Details of these scores are presented in Table 6.

**Table 6:** Prognostic scoring system in status epilepticus: Performance and Components

Scoring tool		Components	Cut-off value for unfavourable outcome	Primary outcome
STESS [81]	Status Epilepticus Status Score	Age ≥ 65 years (2 points) No history of seizures (1 point) GTC (1 point) or NCSE (2 points) Stupor/Coma (1 point)	≥ 3 points [max. 6 points]	In-hospital mortality
EMSE-EACE [54, 56]	Epidemiology-Based Mortality Score in Status Epilepticus (Etiology-Age-Comorbidity-EEG version)	Aetiology (variable) Age (continuous) Comorbidity (CCI, variable) EEG patterns (variable)	≥ 64 points [max. 255 points]	In-hospital mortality
SACE [64]	Salzburg NCSE Criterion A2	Salzburg A2 criteria (1 point) Age < 75 years (1 point) History of seizures (1 point) (NCSE without coma (2 points)	< 3 points [max. 5 points]	In-hospital mortality
END-IT [27]	Encephalitis-Nonconvulsive-Diazepam Resistance-Imaging-Tracheal Intubation Score	Encephalitis (1 point) NCSE (1 point) diazepam resistance (1 point) imaging abnormality (1–2 points) intubation (1 point)	≥ 3 points [max. 6 points]	3-month functional outcome (mRS)
ACD [73]	Age-Consciousness-Duration Score	Age at onset (0–6 points) Consciousness level (0–2 points) SE duration (0–7 points)	Score ≥ 10 points [max. 15 points]	2-year mortality

CCI, Charlson Comorbidity Index; GTC, Generalised tonic–clonic seizures; NCSE, Non-convulsive status epilepticus; SE, Status epilepticus, mRS, modified Rankin Scale, EEG, electroencephalography

## Discussion

This review highlights the evolving understanding of NCSE as a complex neurological emergency that presents significant diagnostic and therapeutic challenges in critical care. The heterogeneous nature of NCSE, with its subtle clinical presentations and diverse underlying aetiologies, underscores the critical importance of maintaining a high index of suspicion, particularly in ICU settings where altered mental status is common. The Salzburg Criteria have introduced a standardised framework [57] which provides clinicians with reproducible tools for identifying NCSE patterns on EEG. However, the persistent challenge lies in distinguishing true epileptic activity from the IIC, particularly in patients with pre-existing encephalopathy. From a therapeutic perspective, management approaches for NCSE highlight important gaps in evidence-based practice. While guidelines provide clear recommendations for first and second-line treatments, the escalation in refractory cases remains largely empirical. Debates surrounding intubation timing and the optimal depth of CIVAD-induced coma reflect the tension between aggressive seizure control and the risk of iatrogenic complications. The finding that 72% of refractory SE patients were successfully managed without intubation challenges traditional approaches and supports more individualised treatment strategies [8].

The emergence of advanced therapeutic options beyond guideline recommendations offers hope for treatment-resistant cases, though the evidence base remains limited. Novel intravenously available ASMs like LCM and BRV show promise, while non-pharmacological approaches such as KD provide additional tools for complex cases.

Prognostic scoring systems have evolved to provide more nuanced outcome predictions, with newer tools like SACE and ACD addressing limitations of earlier models. However, the predominant influence of baseline characteristics over treatment-specific factors on outcomes suggests that early recognition and prompt intervention may be more critical than specific therapeutic choices.

Resource allocation and economic considerations increasingly influence clinical decision-making in NCSE management, particularly regarding the choice between cEEG and rEEG monitoring. Recent data from the Swiss CERTA study demonstrates that while cEEG monitoring does contribute to increased hospital reimbursement costs, the primary driver of healthcare expenses remains length of stay rather than the monitoring modality itself [117]. This contrasts with the United States healthcare system, in which cEEG directly increases hospitalisation charges [37], regardless of clinical outcomes. These findings suggest that the cost-effectiveness of cEEG monitoring may vary significantly across different healthcare systems and billing structures, highlighting the need for region-specific health economic analyses when implementing NCSE diagnostic protocols [128, 129].

Future research priorities should focus on developing biomarkers for early NCSE detection, refining personalised treatment algorithms based on patient-specific factors, and conducting prospective trials to establish optimal management strategies for refractory cases. The integration of cEEG monitoring in high-risk populations and the development of point-of-care diagnostic tools could significantly improve recognition rates and ultimately patient outcomes in this challenging condition.

## Conclusion

NCSE is a common and critical diagnosis requiring dedicated diagnostic and treatment monitoring by experienced ICU and EEG physicians. Due to the common older age and severe comorbidities or existing multimorbidity of NCSE patients, prognosis should be discussed early on with the patient’s relatives or legal guardians. Validated tools are available for the objective prognostication of NCSE. Though less evidence-based than for CSE, different options are available for treatment at any stage of the disease, which should be used in an individualised treatment approach.

## Data Availability

Data sharing is not applicable to this article as no datasets were generated or analyzed during the current study.

## Abbreviations

ACD: Age-Consciousness-Duration
ACNS: American Clinical Neurophysiology Society
AMPA: α-Amino-3-hydroxy-5-methyl-4-isoxazolepropionic acid
ASM: Anti-seizure medication
BID: Two times per day
BIPD: Bilateral independent periodic discharges
BIRDS: Brief potentially ictal rhythmic discharges
BRV: Brivaracetam
BW: Body weight
BZD: Benzodiazepines
CCI: Charlson Comorbidity Index
cEEG: Continuous electroencephalography
CIVAD: Continuous intravenous anaesthetic drugs
CONT: Continuous infusion
CSE: Convulsive status epilepticus
CZP: Cloanzepam
DOC: Disorder of consciousness
DZP: Diazepam
EEG: Electroencephalography
EMSE: Epidemiology-based Mortality Score in Status Epilepticus
END-IT: Encephalitis, NCSE, diazepam resistance, imaging abnormality, tracheal intubation score
ESETT: Established Status Epilepticus Treatment Trial
GABA: Gamma-aminobutyric acid
GPD: Generalised periodic discharges
GPD: Generalised periodic discharges
GRDA: Generalised rhythmic delta activity
GTC: Generalised tonic–clonic seizures
ICU: Intensive care unit
IIC: Ictal-interictal continuum
ILAE: International League Against Epilepsy
INT: Intermittent therapy
IP: Intrapulmonary administration
ISO: Isoflurane
IV: Intravenous
IVIG: Intravenous immunglobulines
KD: Ketogenic diet
KET: Ketamine
LCM: Lacosamide
LEV: Levetiracetam
LPD: Lateralized periodic discharges
LRDA: Lateralized rhythmic delta activity
LZP: Lorazepam
MAC: Minimal alveolar concentration
MDZ: Midazolam
MgSO4: Magnesium sulfate
mRS: Modified Rankin Scale
NCS: Non-convulsive status
NCSE: Non-convulsive status epilepticus
OXC: Oxcarbazepine
PB: Pentobarbital
PER: Perampanel
PHT: (Fos)phenytoin
PLEX: Plasmaexchange
PO: Oral intake
PPF: Propofol
QD: One per day
qEEG: Quantitative electroencephalography
rEEG: Repetitive electroencephalography
RSE: Refractory status epilepticus
SACE: Salzburg Criterion A2 Score
SE: Status epilepticus
SP: Steroid pulse
SRSE: Super-refractory status epilepticus
STESS: Status Epilepticus Severity Score
STP: Stiripentol
SV2A: Synaptic vesicle protein 2A
TERSE: Time-dependent electro-clinical risk stratification
TID: Three times per day
TP: Thiopental
TPM: Topiramate
VPA: Valproate
ZNS: Zonisamide
